# Late Embryogenesis Abundant (LEA) proteins in legumes

**DOI:** 10.3389/fpls.2013.00190

**Published:** 2013-06-25

**Authors:** Marina Battaglia, Alejandra A. Covarrubias

**Affiliations:** Departamento de Biología Molecular de Plantas, Instituto de Biotecnología, Universidad Nacional Autónoma de MexicoCuernavaca, Mexico

**Keywords:** legumes, common bean, soybean, *Medicago*, LEA proteins, water deficit, abiotic stress

## Abstract

Plants are exposed to different external conditions that affect growth, development, and productivity. Water deficit is one of these adverse conditions caused by drought, salinity, and extreme temperatures. Plants have developed different responses to prevent, ameliorate or repair the damage inflicted by these stressful environments. One of these responses is the activation of a set of genes encoding a group of hydrophilic proteins that typically accumulate to high levels during seed dehydration, at the last stage of embryogenesis, hence named Late Embryogenesis Abundant (LEA) proteins. LEA proteins also accumulate in response to water limitation in vegetative tissues, and have been classified in seven groups based on their amino acid sequence similarity and on the presence of distinctive conserved motifs. These proteins are widely distributed in the plant kingdom, from ferns to angiosperms, suggesting a relevant role in the plant response to this unfavorable environmental condition. In this review, we analyzed the LEA proteins from those legumes whose complete genomes have been sequenced such as *Phaseolus vulgaris, Glycine max, Medicago truncatula, Lotus japonicus, Cajanus cajan*, and *Cicer arietinum.* Considering their distinctive motifs, LEA proteins from the different groups were identified, and their sequence analysis allowed the recognition of novel legume specific motifs. Moreover, we compile their transcript accumulation patterns based on publicly available data. In spite of the limited information on these proteins in legumes, the analysis and data compiled here confirm the high correlation between their accumulation and water deficit, reinforcing their functional relevance under this detrimental conditions.

## Introduction

Plants are subjected to different stresses (abiotic and biotic) along their life cycles and have developed different responses to alleviate the damage, and survive to these impaired conditions (Bartels and Sunkar, 2005). Abiotic stresses such as drought, salinity, osmotic, cold, and freezing temperatures produce cellular water deficit, which leads to the accumulation of a group of highly hydrophilic proteins, named LEA proteins (for Late Embryogenesis Abundant) (for review Battaglia et al., 2008; Bies-Etheve et al., 2008; Hundertmark and Hincha, 2008). These proteins are not only associated to water deficit caused by environmental changes but also to water limitation produced during plant development under optimal growth conditions, such as during development of seeds and pollen grains, or some stages of shoot and root development (Colmenero-Flores et al., 1999; Vicient et al., 2000; Sheoran et al., 2006). Some LEA proteins have also been found associated to vascular tissues, and also in meristematic regions (Cheng et al., 1996; Colmenero-Flores et al., 1999; Moreno-Fonseca and Covarrubias, 2001). Their high accumulation in embryos of dormant seeds and in mature pollen grains, both desiccation resistant structures able to withstand dehydration for long periods, led to propose a role for these proteins in plant tolerance to water scarcity [for review see Dure III et al. (1981), Battaglia et al. (2008), Bies-Etheve et al. (2008), Hundertmark and Hincha (2008)].

LEA proteins have been found widely distributed in the plant kingdom, from algae (Honjoh et al., 1995; Joh et al., 1995), moss (Saavedra et al., 2006), ferns (Raghavan and Kamalay, 1993), to angiosperms. Interestingly, LEA-like proteins also accumulate in anhydrobiotic invertebrates and in some bacterial species in response to water limitation (Stacy and Aalen, 1998; Browne et al., 2004; Goyal et al., 2005a; Kikawada et al., 2006; Wang et al., 2007; Hand et al., 2011; Clark et al., 2012).

**Figure 1:**
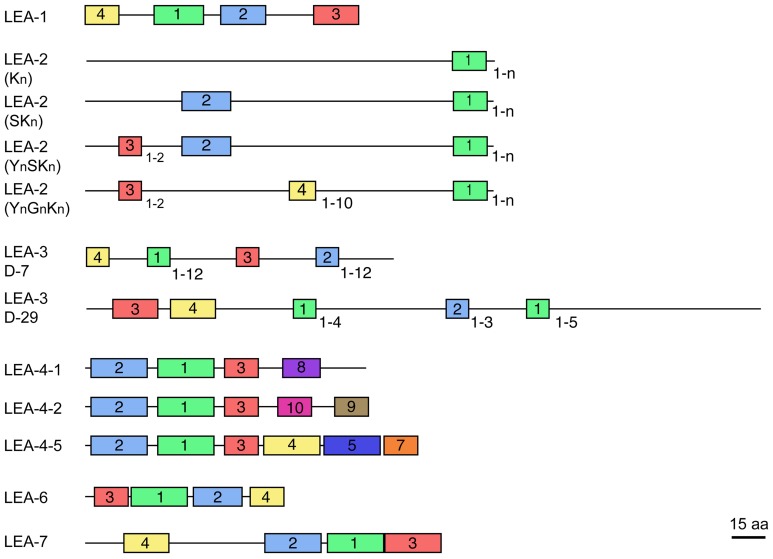
**Schematic representation of motifs distinctive for each LEA protein group (see Table 2)**. The different protein variants found in groups 2, 3, and 4 are shown. Colored blocks indicate the distribution of distinctive motifs in each group; equal colors between groups do not mean sequence similarity. Numbers at the right bottom of some motifs indicate the maximum number of repetitions detected. Protein diagrams are drawn to scale as indicated, considering a mean protein size for each group.

Most LEA proteins identified to date belong to hydrophilins, a widely distributed group of proteins characterized by a high content of charged amino acid residues, as well as glycine or other small amino acids such as alanine, serine, or threonine. Moreover, most hydrophilins lack tryptophanes and cysteines (Garay-Arroyo et al., 2000). These physicochemical properties led to propose that hydrophilins are unstructured proteins in aqueous solutions (Dure, 1993; Garay-Arroyo et al., 2000), which has been shown for some LEA proteins [see for review Olvera-Carrillo et al. (2011)]. Analyses of LEA protein amino acid sequences recognize seven different groups, each one showing distinctive motifs (LEA1 – LEA7). In particular, for groups 2, 3, and 4 different protein variants have been found showing a particular organization of their corresponding motifs (Figure 1). Significant sequence similarity has not been detected between the seven LEA protein groups. Because of their more hydrophobic nature and higher structural order, group 5 LEA proteins are not classified as hydrophilins (Dure III et al., 1989; Battaglia et al., 2008). Given the high heterogeneity and little information on this group, it was not included in this review.

A variety of studies have shown a potential role for LEA proteins in stress tolerance. Their function in protein protection upon water deficit was demonstrated by *in vitro* experiments, where different hydrophilins, including LEA proteins from groups 2, 3, and 4, were able to prevent the inactivation of enzymes such as LDH (lactate dehydrogenase) or MDH (malate dehydrogenase) upon different dehydration levels (Goyal et al., 2005b; Reyes et al., 2005, 2008). Similarly, protective properties were also detected during freeze-thaw *in vitro* assays (Hara et al., 1999; Reyes et al., 2008). Interestingly, in these latter assays, a particular protective role was detected for the K-segment, a LEA2 protein distinctive motif, and for a conserved region in LEA4 proteins (Reyes et al., 2008). Because in these assays, 1:1 ratios between LEA and target proteins were enough to provide protection, it was proposed that this protective activity is carried out by protein-protein interactions (Reyes et al., 2005; Olvera-Carrillo et al., 2011). More severe *in vitro* dehydration assays, using high LEA: target protein ratios, suggested that some LEA and LEA-like proteins are also able to avoid protein aggregation (Goyal et al., 2005b; Chakrabortee et al., 2007). In addition, some reports suggest that the presence of sugars can enhance LEA proteins protecting effect under dehydration (Black et al., 1999; Wolkers et al., 2001; Liu et al., 2010).

Membrane stabilization has been another function attributed to LEA proteins. Most evidence comes from *in vitro* experiments, where LEA2 proteins (dehydrins) were found associated to anionic phospholipid vesicles (Koag et al., 2003, 2009). Also, other assays suggested that dehydrin addition maintain the functional membrane structure under freezing temperatures (Ismail et al., 1999; Kosová et al., 2007). These data are in agreement with reports that have detected binding of dehydrins to membranes or phospholipids; however, all these results have been obtained from *in vitro* experiments or with isolated membranes. In contrast, no conclusive information is available regarding their membrane association by intracellular localization experiments using antibodies or fluorescent translational fusions, which however, have contributed to establish dehydrins location in cytosol, nuclei, chloroplast, protein and lipid bodies, mitochondria, and plasmodesmata [for review Rorat (2006)].

In the case of LEA proteins from other groups, most of them have been shown to be cytosolic; however, some have also been localized in chloroplast (Ndong et al., 2002), mitochondria (Grelet et al., 2005; Tolleter et al., 2010) and even in nucleus (Goday et al., 1994; Houde et al., 1995; Colmenero-Flores et al., 1999; Riera et al., 2004; Gai et al., 2011; Duan and Cai, 2012). The mitochondrial localization of pea (*Pisum sativum*) LEA3 proteins correlates with their ability to protect some mitochondrial enzymes such as rhodanese and fumarase from inactivation induced by *in vitro* dehydration (Grelet et al., 2005). Their nuclei localization has also been associated with binding to DNA for some LEA2, LEA4, and LEA7 proteins (Colmenero-Flores et al., 1999; Maskin et al., 2007; Hara et al., 2009).

Additional roles have been suggested for these proteins such as ion sequestration, in particular, for LEA2 and LEA4 proteins, where histidine-containing motifs seem to be responsible of binding divalent cations (Hara et al., 2005). This is the case of soybean LEA4-5 proteins (GmPM1: Glyma19g32920.1 and GmPM9: Glyma03g30040.1), which bind Fe^+3^, Ni^+2^, Cu^+2^ and Zn^+2^ (Liu et al., 2011). *In vitro* experiments using a citrus dehydrin, or GmPM1 and GmPM9 indicate that these proteins reduce the levels of OH^·^ radical; hence, suggesting an oxidant scavenger activity associated to an increased ion concentration (Hara et al., 2005; Liu et al., 2011).

Over-expression experiments in different organisms have also contributed to support a role of LEA proteins in tolerance to water scarcity. For instance, expression of soybean PM2 protein (LEA3) conferred *Escherichia coli* with the ability to grow under high salt or upon extreme temperature treatments (Liu and Zheng, 2005; Liu et al., 2010). Recently, it has been shown that a LEA3-like protein from the brine shrimp *Artemia franciscana* increases cellular viability after desiccation and hyperosmotic stress of *Drosophila melanogaster* cells (Marunde et al., 2013). In plants, a number of reports indicate that over-expression of LEA proteins from various groups confers tolerance to a variety of water deficit treatments (Puhakainen et al., 2004; Eriksson and Harryson, 2011; Duan and Cai, 2012). More significantly, it has been shown that the deficiency of one or two of the three LEA4 proteins from *Arabidopsis thaliana* is enough to cause water deficit susceptibility (Olvera-Carrillo et al., 2010), showing their relevance in the plant adaptive response to this stress condition. This role is also endorsed for LEA2 proteins as indicated by the co-segregation of a dehydrin gene with chilling tolerance during cowpea (*Vigna unguiculata* L.) seedling emergence (Ismail et al., 1999).

Even though there is very limited information regarding the participation of LEA proteins in the plant response to biotic stress, it is known that over-expression of group 2 LEA proteins from Arabidopsis affects the expression of genes related to plant defense responses (e.g., pathogenesis-related proteins, PR) (Hanin et al., 2011); also, it has been reported that a group 3 LEA protein from maize is up-regulated in response to bacterial infection and that its over-expression in tobacco plants improves tolerance to *Pseudomonas syringae* (Liu et al., 2013). In addition, the over-expression in *Escherichia coli* of groups 2 and 4 LEA proteins causes bacterial growth inhibition (Campos et al., 2006; Hanin et al., 2011). These observations have led to the speculation that the antibacterial activity of some dehydrins may be part of a defense mechanism against opportunistic bacterial infections commonly occurring during water deficit conditions, a hypothesis awaiting exploration.

As it has been described above, the responsiveness of the different *LEA* genes to water deficit is common, however, some of them have also been found to be responsive to other stressful conditions such as ion toxicity, oxidation, or high temperatures, and in some cases a correlation has been found with the ability of LEA proteins to bind different cations, including Fe^+3^, Ni^+2^, Cu^+2^, or to confer tolerance to these stress conditions [for review Hanin et al. (2011)]. Even though these observations may be difficult to integrate, the wide variety of functions that have been attributed to these proteins could be related to their selection throughout evolution not only by low water availability but also by other intrinsically associated conditions; that would be the case of high temperatures or the generation of high ion concentrations and reactive oxygen species, among other effects. In addition, the flexibility of their structure should also be considered, as for many IUPs it has been associated to multifunctionality (Hara, 2010; Liu et al., 2010, 2013; Olvera-Carrillo et al., 2011; Dominguez et al., 2013), an appealing characteristic that has been poorly explored in these proteins.

## LEA proteins in legumes

To gain insight into the conservation patterns of the different distinctive motifs identified in LEA proteins, in this work we analyzed the different legume LEA proteins sequences using the information available in the legume genome sequence databases (*Phaseolus vulgaris:* http://www.phytozome.net/search.php?org=Org_Pvulgaris_v1.0; *Glycine max*: http://www.phytozome.net/search.php?org=Org_Gmax_v1.1; *Medicago truncatula*: http://medicago.jcvi.org/cgi-bin/medicago/overview.cgi; *Lotus japonicus*: http://www.kazusa.or.jp/lotus/), *Cajanus cajan* (http://cajca.comparative-legumes.org/) (Varshney et al., 2012) and *Cicer arietinum* (http://cicar.comparative-legumes.org/) (Varshney et al., 2013) (see Table 1). For this, protein sequences from each group were compared and the motifs described for *Viridiplantae* were analyzed (see Table 2 and Figure 1). In addition to the strong similarity between those motifs previously identified (Battaglia et al., 2008), a new Gly-rich region was found in some legume LEA2 proteins. The most conserved motif in this group is a repetitive 15-mer motif, EKKGIMDKIKEKLPG, called K-segment because of its richness in lysine (K) residues. Additional common motifs are the Y- and S-segments. When present, the S-segment usually precedes the K-segment (Campbell and Close, 1997). The new glycine-rich region detected shows a repetitive large motif represented by the sequence [YF]T[GD]DT[GN][RK]QH[GD]T. This sequence is present up to 10-times in a protein, as in the case of the polypeptide encoded by the *Phvul004G158800.1* gene from *P. vulgaris.* Two dehydrins with Gly-rich motifs have been described. One from *Vigna radiata*, VrDhn1, a novel Y2K dehydrin detected in seeds and induced by various abiotic stresses or ABA treatment (Lin et al., 2012), and Mat1 (Glyma07g10030.1) from soybean, which has a homolog called Mat9 (Glyma09g31740.2), induced upon severe water deficit (Whitsitt et al., 1997; Momma et al., 2003). This motif was only detected in LEA2 proteins from *Phaseoloid* species, which include several legumes more adapted to tropical climates. Because this group is considered one of the youngest groups among the three legume sub-families (Gepts et al., 2005), it can be suggested that this motif is of recent appearance. It is worth mentioning that this motif and its repetitions (4–10X) always were found to be present between segments-Y and -K, which suggests a common ancestor for this type of dehydrins.

**Table 1:**
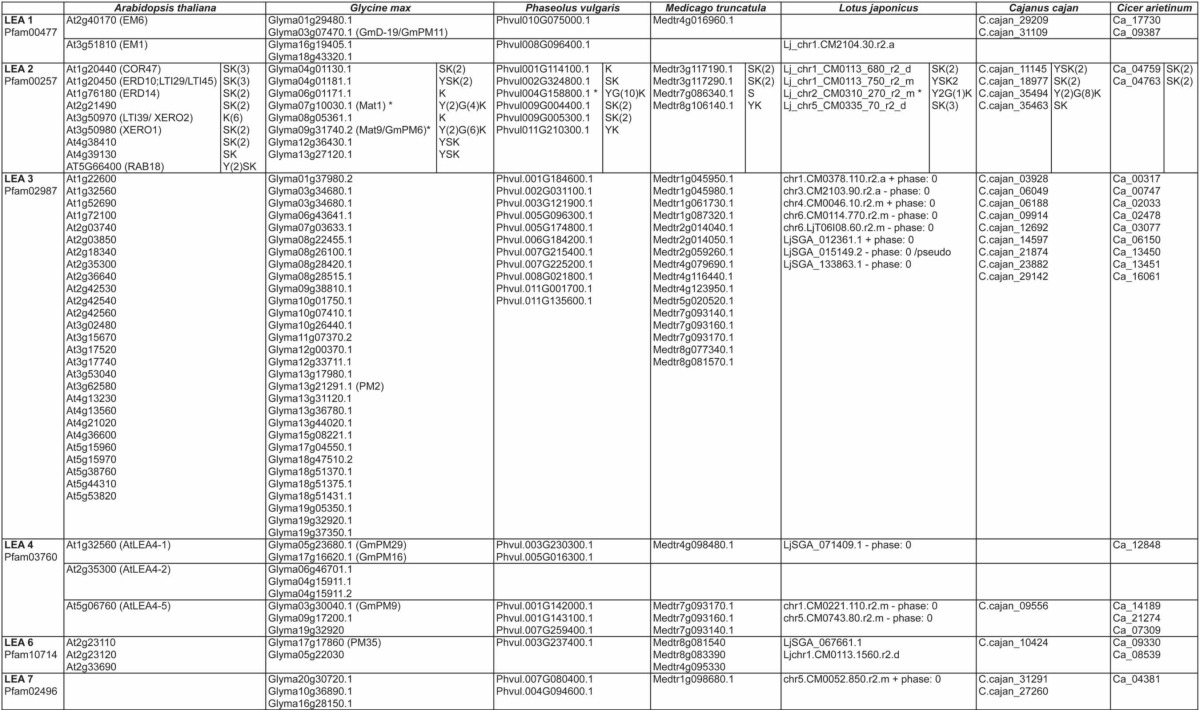
**Legume LEA proteins**.

LEA genes found in different legume genomes and compared with Arabidopsis thaliana genes. Phaseolus vulgaris (http://www.phytozome.net/search.php?org=Org_Pvulgaris_v1.0); Glycine max (http://www.phytozome.net/search.php?org=Org_Gmax_v1.1); Medicago truncatula (http://medicago.jcvi.org/cgi-bin/medicago/overview.cgi); Lotus japonicus (http://www.kazusa.or.jp/lotus/); Cajanus cajan (http://cajca.comparative-legumes.org/) and Cicer arietinum (http://cicar.comparative-legumes.org/). Asterisks indicate LEA2 proteins containing the Gly-rich motif.

**Table 2:**
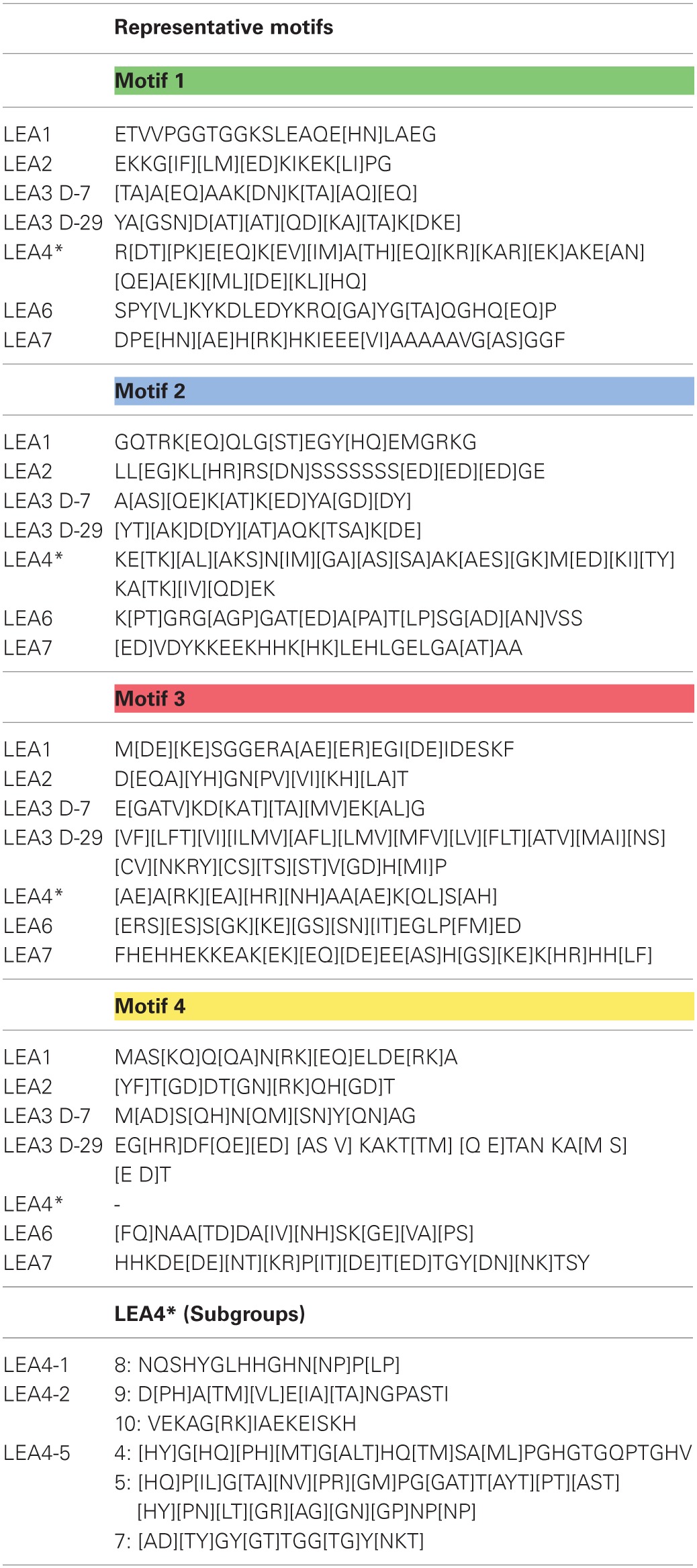
**Distinctive motifs in the different groups of legume LEA proteins**.

**Figure 2:**
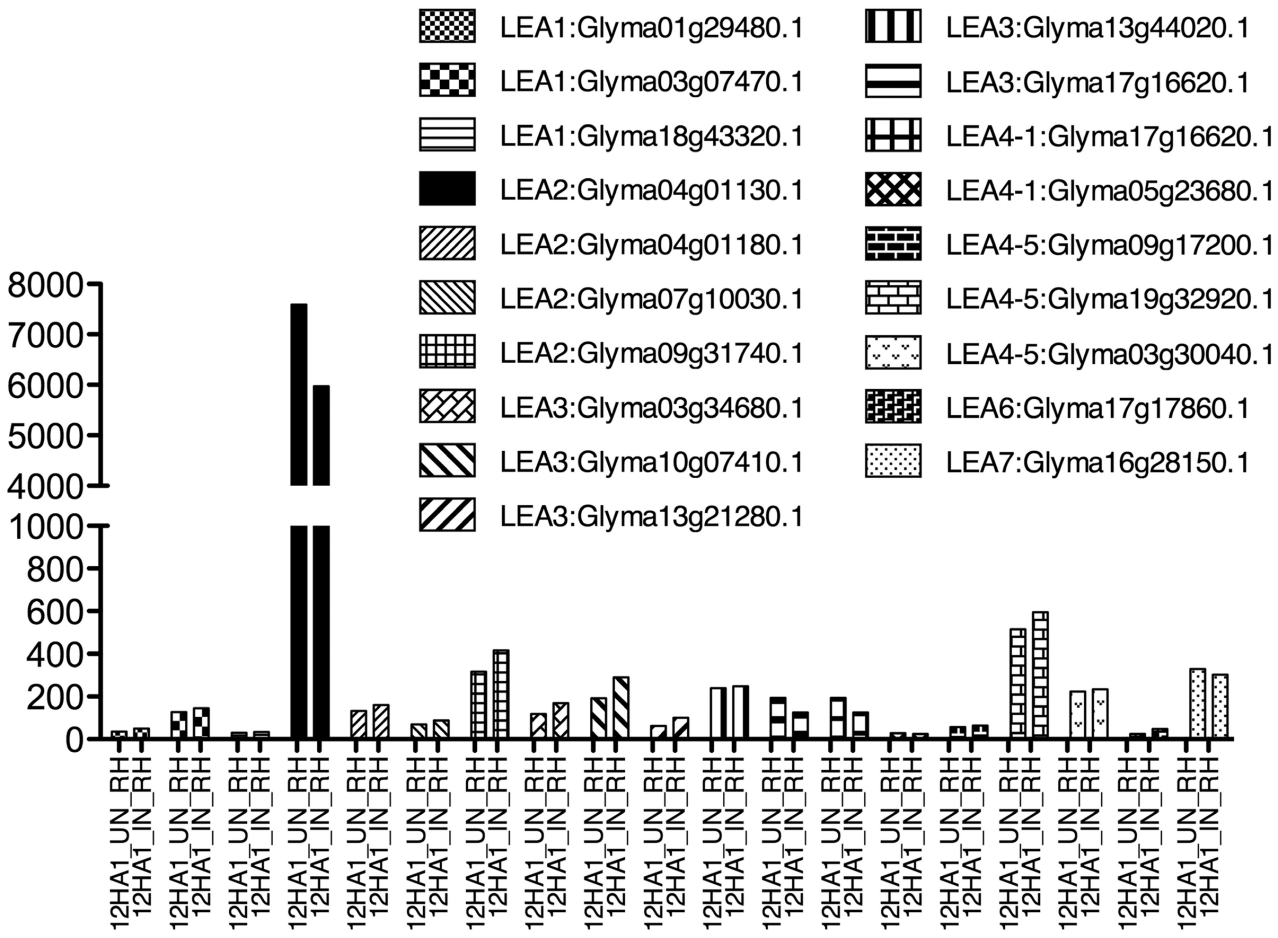
***LEA* gene expression as detected by Libault et al. (2010a), using *Glycine max* RNA isolated from un- and infected-root hair cells harvested after 12 h inoculation**. Infection was done with *B. japonicum*.

LEA protein motifs were discovered using MEME algorithm (4.9.0 version), with an optimum width between 6–25 amino acids, selecting a motif distribution of 0–1 or any number of repetitions (Bailey et al., 2009). The asterisk after LEA4 indicates that for LEA4 proteins, the specific motifs for each sub-group are shown in a separate block. Colors in top bars for every block correspond to those used in Figure 2 for each motif.

### Structure

The LEA protein amino-acid composition predicted structural disorder along their sequence (Dure, 1993; Garay-Arroyo et al., 2000), and thus inscribed them as intrinsically unstructured proteins (IUPs). Structural analyses using circular dichroism (CD) and Fourier transform infrared (FTIR) spectroscopy have shown that this is the case, at least, for some purified recombinant LEA proteins in aqueous solutions (Lisse et al., 1996; Thalhammer et al., 2010). For legume LEA proteins, this has been shown for LEA1 proteins such as GmD19 (Glyma03g07470.1), GmPM11 (Glyma03g07470), LEA2 as GmPM6 (Glyma09g31740.1-LEA2), LEA3 like GmPM30 (Glyma13g44020.1) and LEA4 (Glyma17g16620), where also a potential to attain a more ordered structure by using α-helical inducer agents (i.e., TFE) was observed (Soulages et al., 2002, 2003; Shih et al., 2004, 2010, 2012). In addition, conformational changes could be induced by slow or fast drying that lead to the formation of different α-helical proportions, depending on the protein (Shih et al., 2004, 2012). These results support the idea that unstructured LEA proteins are rather flexible proteins, able to adapt their conformation to the variable cellular environment as that produced by low water availability. It has been proposed that these conformational changes may facilitate interactions between LEA proteins and other macromolecules, such as other proteins, membranes and/or nucleic acids (Boudet et al., 2006; Zhang et al., 2006; Maskin et al., 2007; Battaglia et al., 2008; Hundertmark et al., 2011, 2012; Olvera-Carrillo et al., 2011; Popova et al., 2011).

### Transcript and protein expression patterns

LEA proteins have been detected in different stages of plant development in response to different water deficit conditions (salinity, freezing, and drought). In legumes, this has been reported for common bean, soybean or *Medicago truncatula* seedlings (Blackman et al., 1991; Hsing et al., 1995; Colmenero-Flores et al., 1997; Boudet et al., 2006; Aghaei et al., 2009; Le et al., 2012). As expected, their accumulation during late embryogenesis has also been documented in *M. truncatula* (Kalaiselvi and Manickam, 1999; Chatelain et al., 2012), *G. max* (Shih et al., 2004), *Arachis hypogaea* (Su et al., 2011) and *P. vulgaris* (Colmenero-Flores et al., 1997). Although very little information exists regarding the regulation of the expression of *LEA* genes, for some of them it is known that ABA induced their transcript accumulation (Colmenero-Flores et al., 1997; Bai et al., 2012).

To obtain more information on the accumulation patterns for legume *LEA* genes, we analyzed public microarrays and RNA-sequencing databases (*M. truncatula:*http://mtgea.noble.org/v2/blast_search_form.php; *G. max*: http://soykb.org/; *Lotus japonicus*: http://cgi-www.cs.au.dk/cgi-compbio/Niels/index.cgi?page=i). These data indicate that most legume LEA transcripts and proteins have similar accumulation patterns as those found for LEA proteins from other plant species. *M. truncatula* microarrays showed that water deficit conditions imposed with NaCl treatments (200 mM) induce the accumulation of transcripts from *LEA* genes of groups 2, 3, 4, 6, and 7. *LEA1* transcripts were mostly detected during late embryogenesis as in *Arabidopsis* (Manfre et al., 2006, 2009), cotton (Dure III et al., 1989), and wheat (Litts et al., 1987, 1991). *M. truncatula* proteome analysis showed that MtEm6 protein is particularly present in newly emerged root tips (Zhang et al., 2006), as well as its transcript, as showed by microarray data.

### Their presence in meristematic regions and root hairs

Interestingly, scrutiny of soybean microarray information showed that *LEA* transcripts from different groups accumulate in root tips and apical meristems, however, this just applies to some of them. For example, Glyma03g07470.1 (LEA1) was only detected in root tips, as did Glyma04g01130.1 (LEA2), and Glyma16g28150.1 (LEA7). All *LEA3* and *LEA4* transcripts accumulate in root tips except Glyma10g26440.1 (LEA3), Glyma17g04550.1 (LEA3), and Glyma09g17200.1 (LEA4). Accumulation in apical meristems was detected for Glyma04g01130.1 (LEA2), Glyma17g16620.1 (LEA3), and Glyma13g44020.1 (LEA3), all LEA 4 except Glyma05g23680.1, and all *LEA7* transcripts. While soybean *LEA6* transcripts were not detected in meristems, the transcript and protein for its homologous gene accumulate in vegetative and root meristems from principal and lateral roots of *P. vulgaris* (Colmenero-Flores et al., 1999; Moreno-Fonseca and Covarrubias, 2001). These observations strongly support the idea that LEA proteins could protect stem cells niches from changes in environmental water availability preventing and/or avoiding damage to these cells vital for plant survival.

A group of root epidermal cells, the atrichoblasts, has a specific cell fate to develop and differentiate as root hairs; they are formed by one tubular cell with polar growth (Cárdenas, 2009). Root hairs play a crucial role in plants, because water and nutrient uptake from soil occur principally through them. In legumes, root hairs have an additional role, they are the cells that interact, recognize and constitute the entrance of their symbiont, the rhizobial bacteria (Goormachtig et al., 2004). In these cells, LEA proteins have been detected in non-legumes growing under optimal irrigation conditions, as shown for *AtEm6*, a *LEA1* gene (Vicient et al., 2000). Legume *LEA* transcript accumulation in root hairs was analyzed from a public database generated by comparing soybean transcriptome sequences obtained from free—or *B. japonicum—*inoculated root hairs (Libault et al., 2010a). Interestingly, *LEA* transcripts from all groups were found to accumulate in root hairs of non-infected plants (Figure 2), such as *LEA2* (Glyma04g01130.1, Glyma04g01180.1, Glyma07g10030.1, and Glyma09g31740.1), *LEA3, LEA4* (all), *LEA6* (Glyma17g17860.1), and *LEA7* (Glyma16g28150.1); however, none of them showed higher accumulation by rhizobia infection. Analysis of a proteome from isolated root hair cells identified the accumulation of only three LEA proteins: Glyma13g31120; Glyma03g34680 (LEA3), and Glyma04g01130.1 (LEA2), most probably due to the limitations inherent to the sensitivity of detection and protein extraction methods, and the properties of the proteins.

### Accumulation in nodules

**Figure 3:**
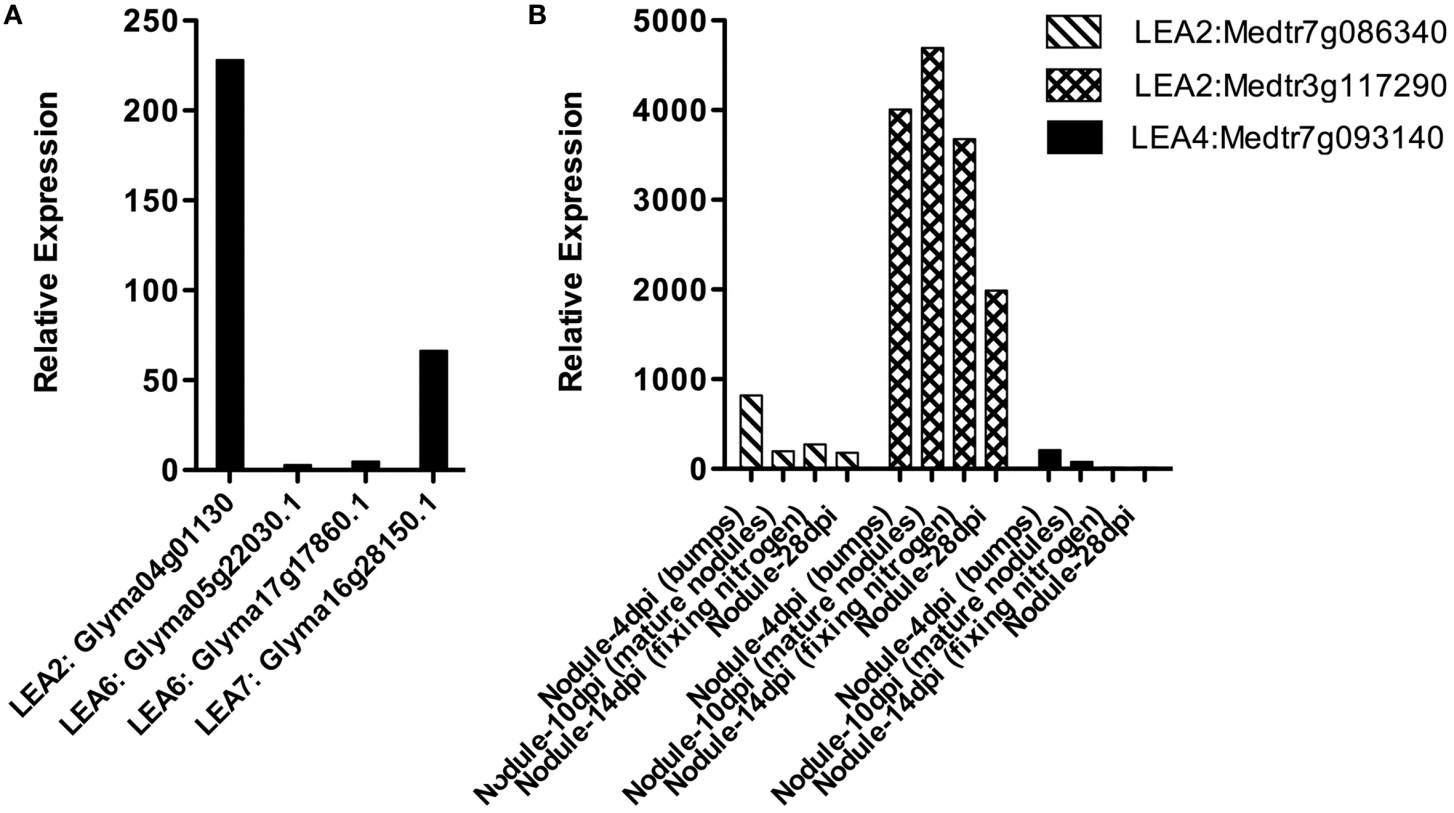
***LEA* gene expression patterns obtained using RNA isolated from *Glycine max***(A)** or *Medicago truncatula***(B)** nodules**. (Benedito et al., 2008; Libault et al., 2010b; Severin et al., 2010; Joshi et al., 2012). dpi: days post-infection.

Nitrogen fixation is one of the fundamental processes in nature, carried out by N_2_ fixing-organisms. In some cases, this activity is possible because the ability of some bacteria to establish symbiosis with legumes, a process that implies a perfect coordination between the plant and the bacteria and that results in the formation of a new organ, the nodule, where nitrogen fixation occurs. *Rhizobium* is a bacterial group that by means of a fine molecular communication detects, recognizes and establishes symbiosis with plants. In the nodule, rhizobia develop as bacteroids that will be the N_2_ fixation competent bacterial stage (Oldroyd and Downie, 2008). Because the relevance of nodules in this function, the accumulation of LEA proteins and their transcripts was investigated in these organs from the *M. truncatula* Gene Expression Atlas (MtGEA) Project (Benedito et al., 2008) and *G. max* RNA-Seq atlas (Libault et al., 2010b; Severin et al., 2010), where some expression patterns are available. The results showed that *LEA* genes are expressed in nodules; however, we did not find representatives for all groups (Figure 3). LEA1 and LEA3 transcripts were not detected in these two legumes. The identified *LEA* transcripts corresponded to group 2 (*Medtr7g086340.1, Medtr3g117290.1*, and *Glyma04g01130*), group 4 (only one from *M. truncatula, Medtr7g093140.1- LEA4-5* in 4 dpi nodule primordia), group 6 (*G. max LEA6 genes*), and group 7 (only one gene from *G. max, Glyma16g28150.1*).

We found differences between the expression of *LEA* genes in *M. truncatula* and *G. max* and also between the different groups of LEA proteins (see Figure 3). LEA2 transcripts were detected in both species for genes *Medtr7g086340.1, Medtr3g117290.1*, and *Glyma04g01130*. Only one LEA4 protein was detected in primordia (4 days after inoculation) of *M. truncatula* (Medtr7g093140.1- LEA4-5), while all LEA6 genes from *G. max* were detected in nodules. From group 7 only one gene (Glyma16g28150.1) was found. A significant increase in the accumulation of two group 2 LEA protein transcripts (*GmLEA8 and GmLEA10*) has also been detected in soybean roots after 35 days of inoculation with *Bradyrhizobium japonicum* in well-watered plants; unexpectedly, lower accumulation levels of both transcripts were found in drought-stressed plants after the same inoculation time, suggesting that these LEA proteins may play a role during *B. japonicum* infection, induced by the “stress” caused during this process and/or by some developmental cue. The accumulation patterns for these transcripts have been also obtained from the corresponding nodules, where transcripts were not detected in well-irrigated plants, while nodules from drought-stressed plants, as expected, showed increased accumulation levels for both *LEA* mRNAs (Porcel et al., 2005). The response of these two genes (*GmLEA8 and GmLEA10*) was also examined in soybean plants colonized by the arbuscular mycorrhizal fungus *Glomus mosseae*, grown under optimal irrigation or stress conditions. Even though, roots of inoculated drought-stressed plants showed high accumulation of these transcripts, the accumulation levels were lower than non-inoculated stressed plants, supporting the idea that the association with mycorrhiza brings on a plant tolerance response possibly due to changes in root morphology produced by this symbiosis. Accordingly, no expression was detected upon mycorrhiza inoculation in contrast to the accumulation response to *B. japonicum* infection (Porcel et al., 2005).

## Concluding remarks

LEA proteins have represented for a long time an enigma in plant biology. The lack of similarity to other proteins of known function and their high structural flexibility has hampered the progress regarding their activity and the implicated mechanisms. However, the high association between their accumulation and different levels of water deficit, caused during development or by the environment, has contributed to build hypotheses regarding their role in the plant tolerance to those conditions that produce low water availability, as drought, salinity, or extreme temperatures.

This has led to relevant advances concerning their transcript and protein accumulation and expression patterns in response to abiotic stress or throughout development, their participation in these processes, and their structural properties. The available information presents LEA proteins as a set of proteins whose participation in plant tolerance to water deficit conditions has gained experimental support, and also as proteins whose flexible structure turns them as a good model to find the functional relevance of this flexibility in the plant response to stress.

Because most of the information regarding LEA proteins has been obtained from Arabidopsis, in this work we have focused on relevant data reported from legumes, to explore whether the observations made in Arabidopsis can be extended to this family of plants, or whether legume LEA proteins present some distinctive structural or functional features.

The analysis of the compiled data from literature and from the available databases showed that most of the structural characteristics recognized in these proteins are conserved in their corresponding homologs in the different legume LEA protein groups; however, it also revealed an additional motif in LEA2 proteins not detected to date in other plant families. It is worthwhile mentioning that LEA7 proteins, a group not present in Arabidopsis but detected in many other species, is also in legumes. As expected, the transcript and protein accumulation patterns available show their responsiveness to water deficit imposed by salinity, high osmolarity, dehydration, and low temperatures, supporting their participation in legume responses to these stressful conditions.

Of particular interest was the finding that various *LEA* transcripts from all groups accumulate in meristematic regions, even in plants grown under optimal growth conditions supporting a protective role in these fragile but critical regions for plant adaptation and survival, as proposed by Olvera-Carrillo et al. (2010), given that the over-expression or deficiency of a LEA4 protein led to an increase or a reduction, respectively, in the number of floral and axillar buds in Arabidopsis plants subjected to water deficit treatments. Moreover, different *LEA* transcripts were also detected in root hairs, which as in the previous case are frail but essential organs not only for water absorption but also to establish symbiosis between legumes and rhizobia needed for nitrogen fixation. Nodules also contain some LEA proteins; however, not all LEA protein groups seem to be present in the different types of nodules. A more detailed analysis is required, where the transcript levels of LEA protein from different groups is determined considering different stages during nodule development. With this limited and heterogeneous information it is difficult to be certain of the absence of some LEA protein groups in nodules. Similarly, at this point proposing tentative roles of these proteins during nodule development under optimal conditions would be highly speculative.

The information in this work regarding LEA proteins in legumes exposes the relevance of these proteins in the response and adaptation of this family of plants to water limiting environments, as it has been shown in other species, and justifies further studies to understand their function and participation in the plant responses to stress and during development. Also, it opens new interesting avenues regarding their role in meristematic regions and other fragile organs, such as root hairs, of particular importance in legumes symbiosis. All this predicts an appealing scenario full of surprises and knowledge.

### Conflict of interest statement

The authors declare that the research was conducted in the absence of any commercial or financial relationships that could be construed as a potential conflict of interest.

## Abbreviations

Groups of LEA proteins: LEA1: LEA2, LEA3, LEA4, LEA6, and LEA7.
